# Smoking behavior is associated with suicidality in individuals with psychosis and bipolar disorder: a systematic quantitative review and meta-analysis

**DOI:** 10.3389/fpsyg.2024.1369669

**Published:** 2024-09-12

**Authors:** Jakob Pietschnig, Sandra Oberleiter, Marcel D. Köhler

**Affiliations:** ^1^Department of Developmental and Educational Psychology, Faculty of Psychology, University of Vienna, Vienna, Austria; ^2^Wiener Werkstaette for Suicide Research, Vienna, Austria

**Keywords:** smoking, suicide, meta-analysis, psychosis, quantitative review, bipolar disorder

## Abstract

Smoking behavior has been well-established to be more prevalent in individuals with psychosis and bipolar disorder compared to the general population. However, reports about higher suicide attempt prevalence of smoking compared to non-smoking patients suggest that smoking behavior may contribute to identifying at-risk groups of patients in a comparatively easy manner. In the present systematic quantitative review, we provide meta-analytical evidence on the smoking and suicide attempt link in 22 studies (*k* = 27 independent samples; *N* = 11,452) of patients with psychosis and bipolar disorder. We observed a small meaningful effect of smoking on suicide attempts (*OR* = 1.70; 95% *CI* [1.48; 1.95]), indicating that smokers have 1.70 the odds of having reported a suicide attempt compared to non-smokers. This effect generalized across diagnosis type (i.e., schizophrenia vs. bipolar spectrum disorder), sample type (i.e., in-vs. outpatients), and participant sex. However, the observed summary effect appeared somewhat inflated due to publication process-related mechanisms, showing some evidence for effect-inflating publication bias and a decline effect. In all, the presently observed smoking and suicide attempt link appears to be small but meaningful and robust, thus suggesting smoking status represents a useful variable for the identification of at-risk populations for suicide attempts.

## 1 Introduction

It has been well-established that people who have been diagnosed with mental illnesses have elevated smoking rates (Hartz et al., 2014). For instance, psychotic patients have been reported to show a smoking prevalence that is more than twice as large compared to that of the general population (Dickerson et al., 2013). However, individuals with psychosis are not only more likely to smoke but also to consume more, stronger, and often unfiltered cigarettes (and therefore more laden with nicotine and tar) (de Beaurepaire et al., 2012). Moreover, they have been reported to have more difficulty quitting than other individuals (Haustein et al., 2002).

Several candidate theories have been proposed that may explain the smoking prevalence differences between healthy adults and individuals diagnosed with psychosis and bipolar disorder. Arguably, the most prominent theory is currently the self-medication theory (Isuru and Rajasuriya, 2019), which postulates that psychotic and bipolar patients typically show predating substance abuse (i.e., before the onset of the mental condition), a more substantial nicotine addiction than general population samples, and an ongoing benefit (e.g., counteracting medical side effects, improving cognition) regarding nicotine consumption (Kumari and Postma, 2005). Other potential explanations postulate a shared diathesis (e.g., higher risk of developing mental illnesses and nicotine dependence due to shared genetic risk factors) between smoking and psychosis (Khokhar et al., 2018) or a unidirectional causal influence of nicotine on the development of mental illness (Gurillo et al., 2015).

For individuals with psychosis and bipolar disorders, both smoking and previous suicide attempts have been suggested to represent risk factors that put them at a considerably higher risk for suicide than the general population (Cassidy et al., 2018; Pompili et al., 2013). The self-medication theory posits that problem-solving difficulties in people diagnosed with psychosis and bipolar disorder, which include emotional processing, social clue perception, attributional style, theory of mind, and the sharing, understanding, and response to others’ emotions (Bora et al., 2009; Green et al., 2015; Savla et al., 2013), could be alleviated through the activation of nicotinic acetylcholine receptors by tobacco in the cerebral cortex area (Gil and Metherate, 2019). On the one hand, smoking seems to improve cognitive functions, such as problem-solving and attention (Knapp et al., 2017; Lee and Van Meter, 2020; Martin and Sayette, 2018; Miskowiak et al., 2019). On the other hand, these beneficial effects are comparatively short-lived, and improvements fade quickly.

Consistent with this idea, some studies have shown that suicide risk appears to be elevated in smokers with psychosis and bipolar disorder. Previous meta-analytical accounts on the smoking and suicide ideation link suggested positive associations in the general population (e.g., Sankaranarayanan et al., 2015) as well as in individuals with psychosis (Pietschnig et al., 2019). In fact, (i) seemingly similar patterns of the smoking and suicidality link that have been reported in past studies for both patients with psychosis and bipolar disorders as well as (ii) common proposed candidate mechanisms that these patterns are rooted in may be interpreted as evidence for potentially similar associations between smoking and suicidality in these patient groups.

However, the strength and meaningfulness of this effect in patients with psychosis and bipolar disorder remains to date unclear. Low sample numbers and indications of potentially confounding effects of dissemination bias in a previous meta-analysis on this topic (Sankaranarayanan et al., 2015), as well as the decline effect (i.e., a larger likelihood of effect strengths to decrease rather than to increase over time, e.g., Pietschnig et al., 2019), warrant an update of the existing evidence. In the present systematic review, we examine all available evidence of the smoking and suicidality link in individuals diagnosed with psychosis and bipolar disorder while accounting for potential influences of dissemination bias.

## 2 Methods

The present study was preregistered prior to accessing the data. The preregistration protocol, deviations from the preregistration, and PRISMA Checklist are available on the Open Science Framework (OSF; https://osf.io/j69dk and https://osf.io/yxn2k/).

### 2.1 Literature search

The literature search was concluded in January 2024. We searched five databases for eligible published studies (ISI Web of Science, PubMed, PsychINFO, CINAHL, and Scopus). Furthermore, the Open Access Dissertation and Theses database (oadt.org) was searched for grey literature. We used the following search string: (“smok* OR “nicotine”) AND (“suicide*” OR “suicide attempt”) AND (“psychosis” OR “psychotic” OR “bipolar” OR “schizophren*”). In August 2024, the literature search was updated and the search string was extended to include the search term “tobacco use.”

### 2.2 Inclusion criteria

**Figure 1:**
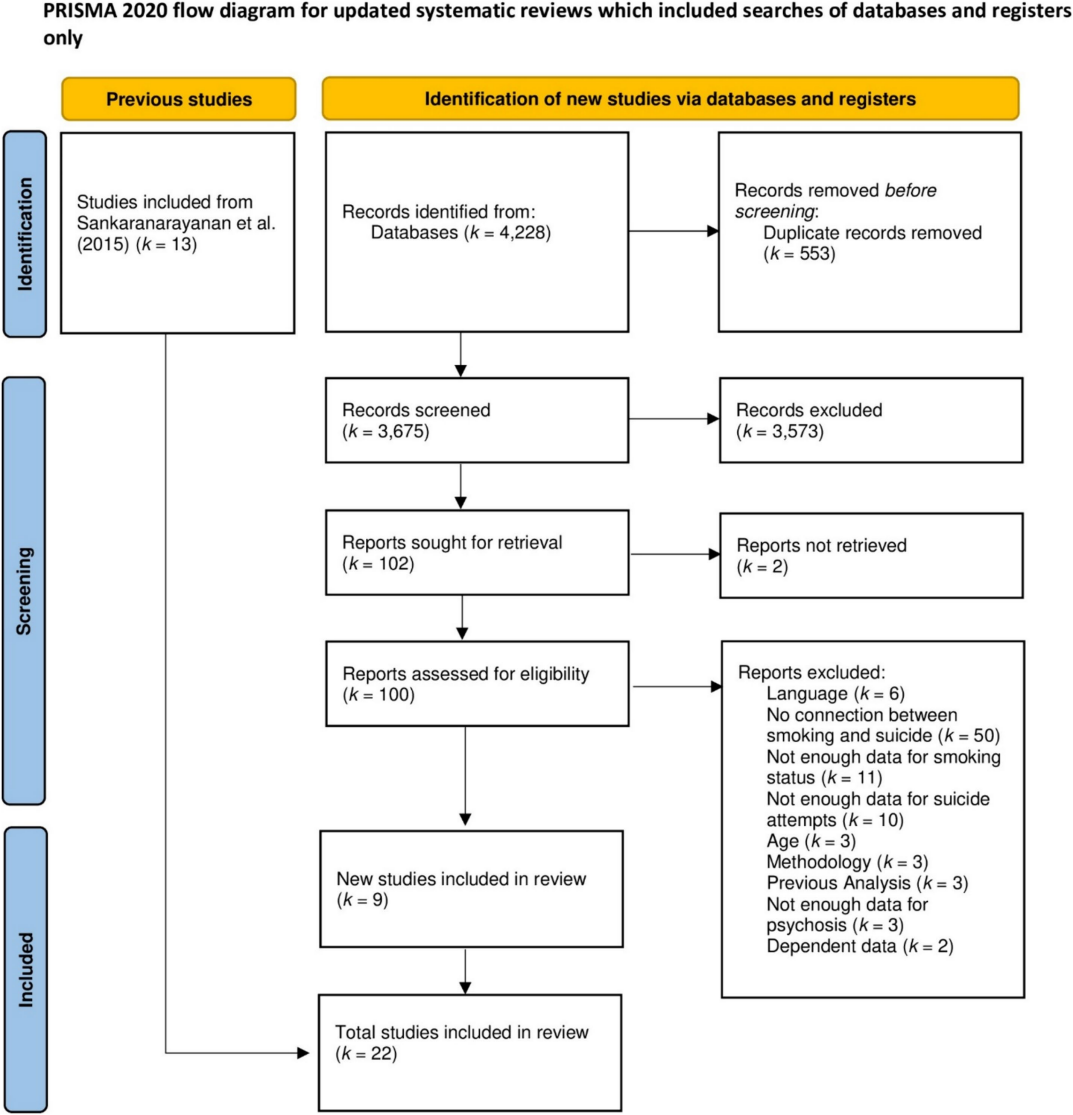
PRISMA-flow chart for study inclusion.

To be included in the present meta-analysis, studies had to meet seven inclusion criteria. They had to (i) be observational, (ii) provide an odds ratio or sufficient statistical parameters to calculate one for smoking and suicide attempts, (iii) include lifetime or past year reports of suicide attempts, (iv) report the respective sample size, (v) report data of people with psychotic disorders (i.e., schizophrenia, schizoaffective disorder, first-episode psychosis, delusional disorder, bipolar disorder, or psychotic depression), (vi) comprise adult participants (mean sample age = 18 years+), and (vii) be published in English. In all, 3,603 study titles and abstracts were screened after duplicate removal (see Figure 1 for a PRISMA flow chart). Study coding was conducted twice independently by one researcher. Discrepancies were resolved by discussion with another independent and experienced researcher. All data are provided on the OSF.^1^

### 2.3 Data extraction and coding

We coded event vs. non-event data (i.e., numbers of smokers and non-smokers who did or did not attempt suicide) into categories and recorded study-specific variables: Study type (cohort vs. cross-sectional vs. case–control); sample type (inpatients vs. outpatients vs. mixed); diagnosis type (schizophrenia spectrum vs. bipolar spectrum vs. mixed; of note, a further differentiation according to type of diagnosis was not feasible due to low study cell counts in terms of reported diagnoses); sample size; country of publication. In cases of missing information, data were requested from corresponding authors by email. If no responses were received, individual entries were coded as missing.

### 2.4 Primary study quality assessment

We used the Newcastle-Ottawa Quality Assessment Scale (Wells et al., 2021) to assess study quality regarding risk of bias assessments according to an adapted approach for cross-sectional studies (Dürlinger and Pietschnig, 2022). Primary study quality was assessed by an experienced researcher [J.P.] and rated according to four criteria (ratings are provided at https://osf.io/brzs4). Rating sum scores were subsequently correlated with primary study effect sizes to assess potential influences of study quality on effect size estimation. No statistically significant influences of primary study quality ratings on effect estimation were identified in the present study in a precision-weighted meta-regression (*Q* = 1.78; *p* = 0.182).

### 2.5 Statistical analyses

We used random-effects models to calculate summary effects. For our calculations, odds ratios were transformed into log odds ratios and, after analysis, back-transformed for ease of interpretation. All analyses were conducted with the open source software R 4.10 (R Core Team, 2023) [packages: metafor (Viechtbauer, 2010), data.table (Dowle et al., 2019), esc (Lüdecke et al., 2019), puniform (van Aert et al., 2020), readxl (Wickham et al., 2019), tidyverse (Wickham and Wickham, 2017), and weightr (Coburn et al., 2019)]. Between-studies heterogeneity was interpreted according to well-established thresholds of the *I*^2^-index [i.e., 25, 50, and 75% representing lower thresholds of small, moderate, and large heterogeneity, respectively; (Higgins et al., 2003)]. Following well-established benchmarks, Odds Ratios of 1.44, 2.48, and 4.27 (corresponding to log*OR* = 0.36, 0.91, and 1.45, respectively) were interpreted as the lower thresholds of small, medium, and large effects (Cohen, 1988).

### 2.6 Moderator analyses

We used mixed-effects subgroup analyses to assess potential influences of categorical moderators, specifically, diagnosis type (bipolar vs. schizophrenia vs. mixed), sample type (inpatients vs. outpatients vs. mixed), and study type (case–control vs. cohort vs. cross-sectional). We used precision-weighted mixed-effects meta-regressions to assess the potential effects of the continuous variables sex (percentage of women within samples) and publication year.

### 2.7 Publication bias

To detect potential influences of confounding dissemination bias, we used several different bias detection approaches (i.e., visual funnel plot inspection, formal funnel plot asymmetry-based assessments, selection model assessments, and *p*-value-based methods), following current recommendations (Siegel et al., 2022). First, we visually inspected funnel plots for potential asymmetry. Second, Sterne and Egger’s regression test (Egger et al., 1997) and Trim-and-Fill (Duval and Tweedie, 2000) were applied. The Sterne and Egger regression regresses effect standard deviates (i.e., standardized effects) on study precision. A significant intercept can be interpreted as evidence of publication bias. In Trim-and-Fill, funnel plot asymmetry is assessed and excessive effects in terms of strength according to the estimated summary effect are trimmed. Subsequently, the summary effect is re-estimated according to the trimmed data set. This iterative procedure is repeated until no further asymmetry is detected. Then, trimmed studies are reinserted, and presumably missing effects are symmetrically imputed on the opposite side of the funnel. Notably, the resulting adjusted effect size should not be seen as a corrected estimate but should be rather interpreted in the sense of a sensitivity analysis.

Third, we examined the effects of a moderate one-tailed selection based on Vevea and Woods (2005) selection model approach. In this method, effect sizes are weighted according to the publication likelihood of specific assumed *p*-values (we followed a standard weighting scenario as suggested by the authors). Their distribution is then compared to an unweighted model. The resulting effect estimates should be similar in the absence of publication bias.

Finally, we used *p*-uniform (Van Assen et al., 2015) and *p*-curve (Simonsohn et al., 2014) to assess potential bias. Both methods are based on the idea that study *p*-values are uniformly distributed in the presence of a null effect. With increasing non-null effect strength, the *p*-value distribution becomes increasingly right-skewed. An identical phenomenon should be observable when focusing exclusively on *p*-values that fall below the typically assumed significance threshold of 0.05. In *p*-curve, formal tests allow an assessment (i) of the evidential value of a given set of study effects as well as (ii) effect-distorting effects of *p*-hacking (i.e., extensive use of questionable research practices) based on the observed *p*-value distributions.

In *p*-uniform, conditional distributions of significant *p*-values based on the corresponding population effect size are compared with a uniform distribution to assess evidence for *p*-hacking. Moreover, this approach allows a summary effect and confidence interval estimation based only on the observed significant *p*-values and their associated degrees of freedom. Following current recommendations, we interpreted *p*-values <0.10 or differences between estimated effects exceeding 20% as indicative of publication bias in all detection methods (Siegel et al., 2022).

### 2.8 Final sample

We included 22 studies comprising *k* = 27 independent effect sizes (*N* = 11,452 patients; mean sample age = 39.5 years). Most participants were schizophrenic spectrum (*n* = 4,797) and bipolar spectrum patients (*n* = 4,020), with the remaining samples comprising both diagnosis types (*n* = 2,635). The majority of samples comprised outpatients (*n* = 6,269), with the remaining samples consisting of inpatients (*n* = 2,566) and mixed patient groups (*n* = 2,590). Primary study characteristics are detailed in Table 1.

**Table 1:** Characteristics of included studies according to published reports.

Study (year)	*N*	Smokers		Non-smokers		*OR*	*LBCI*	*UBCI*	Study type	Diagnosis type	Mean age	% Women	Sample type	Country
		Suicide attempts	No suicide attempts	Suicide attempts	No suicide attempts									
Altamura et al. (2003)	103	20	54	2	27	–	–	–	Cohort	SCZ	39.1	33.98	Out	Italy
Jarbin et al. (2004)	41	8	10	2	21	–	–	–	Cohort	SCZ	37.1	46.34	Out	Sweden
Jarbin et al. (2004)	33	4	4	4	21	–	–	–	Cohort	BP	37.1	57.58	Out	Sweden
Ostacher et al. (2006)	399	–	–	–	–	2.74	1.77	4.23	Cohort	BP	38.6	54.14	In	United States
Iancu et al. (2006)	61	21	16	11	13	–	–	–	Case	SCZ	41.2	40.98	Mx	Israel
Altamura et al. (2007)	400	262	22	97	19	–	–	–	Cross	SCZ	37.2	30.5	Out	United States and Canada
Altamura et al. (2007)	232	131	38	41	22	–	–	–	Cross	SCZ	36.4	39.23	Out	France, Italy, United Kingdom
Altamura et al. (2007)	198	96	19	60	23	–	–	–	Cross	SCZ	38.1	53.03	Out	Czech, Hungary, Croatia
Altamura et al. (2007)	37	20	4	11	2	–	–	–	Cross	SCZ	35.3	37.84	Out	South Africa
Altamura et al. (2007)	93	57	6	25	5	–	–	–	Cross	SCZ	36.7	41.94	Out	Argentina and Chile
Baethge et al. (2009)	352	44	116	30	162	–	–	–	Cohort	BP	27.7	50.85	Out	Italy
Ostacher et al. (2009)	116	5	26	3	82	–	–	–	Cohort	BP	42	60.34	Out	United States
Andriopoulos et al. (2011)	106	8	71	0	27	–	–	–	Case	SCZ	27.9	30.19	Out	Greece
Kao et al. (2011)	95	40	22	13	20	–	–	–	Cross	SCZ	40.1	52.63	Out	Taiwan
Gutiérrez-Rojas et al. (2012)	108	–	–	–	–	5.4	1.9	15.5	Cross	BP	–	–	Out	Spain
Baek et al. (2013)	1,643	195	530	167	751	–	–	–	Cross	BP	40.2	68.65	Out	United States
Kanwar et al. (2013)	790	–	–	–	–	1.53	1.11	2.11	Cross	SCZ	–	–	Mx	Germany
Sankaranarayanan et al. (2014)	1,812	628	579	276	329	–	–	–	Cross	MX	38.4	40.51	Out	Australia
Ducasse et al. (2015)	453	88	104	91	170	–	–	–	Cross	BP	42.3	56.07	Out	France
Antypa et al. (2016)	553	133	173	80	167	–	–	–	Cross	MX	47.4	61.84	Mx	Belgium
Xia et al. (2018)	300	13	47	25	215	–	–	–	Case	SCZ	28.3	61.33	In	China
Dickerson et al. (2019)	270	77	40	79	74	–	–	–	Cross	MX	38.7	46.3	Mx	United States
Icick et al. (2019)	916	150	247	188	331	–	–	–	Case	BP	40.7	59.17	Mx	Norway and France
Mallet et al. (2019)	474	18	241	9	206	–	–	–	Cross	SCZ	32.2	24.26	Out	France
Dai et al. (2020)	906	71	531	26	278	–	–	–	Cross	SCZ	46.4	18.32	In	China
Liu et al. (2020)	767	81	394	44	248	–	–	–	Case	SCZ	–	18.25	In	China
Dai et al. (2021)	194	15	82	13	84	–	–	–	Cross	SCZ	46.5	0	In	China

LBCI, Lower bound of 95% confidence interval; UBCI, Upper bound of 95% confidence interval; cohort, cohort study; cross, cross-sectional study; case, case–control study; SCZ, patients with schizophrenia; BP, patients with bipolar disorder; MX, mixed patient groups; in, inpatients; out, outpatients; mx, mixed sample of in-and outpatients; references of included studies are available at https://osf.io/beh54.

## 3 Results

**Figure 2:**
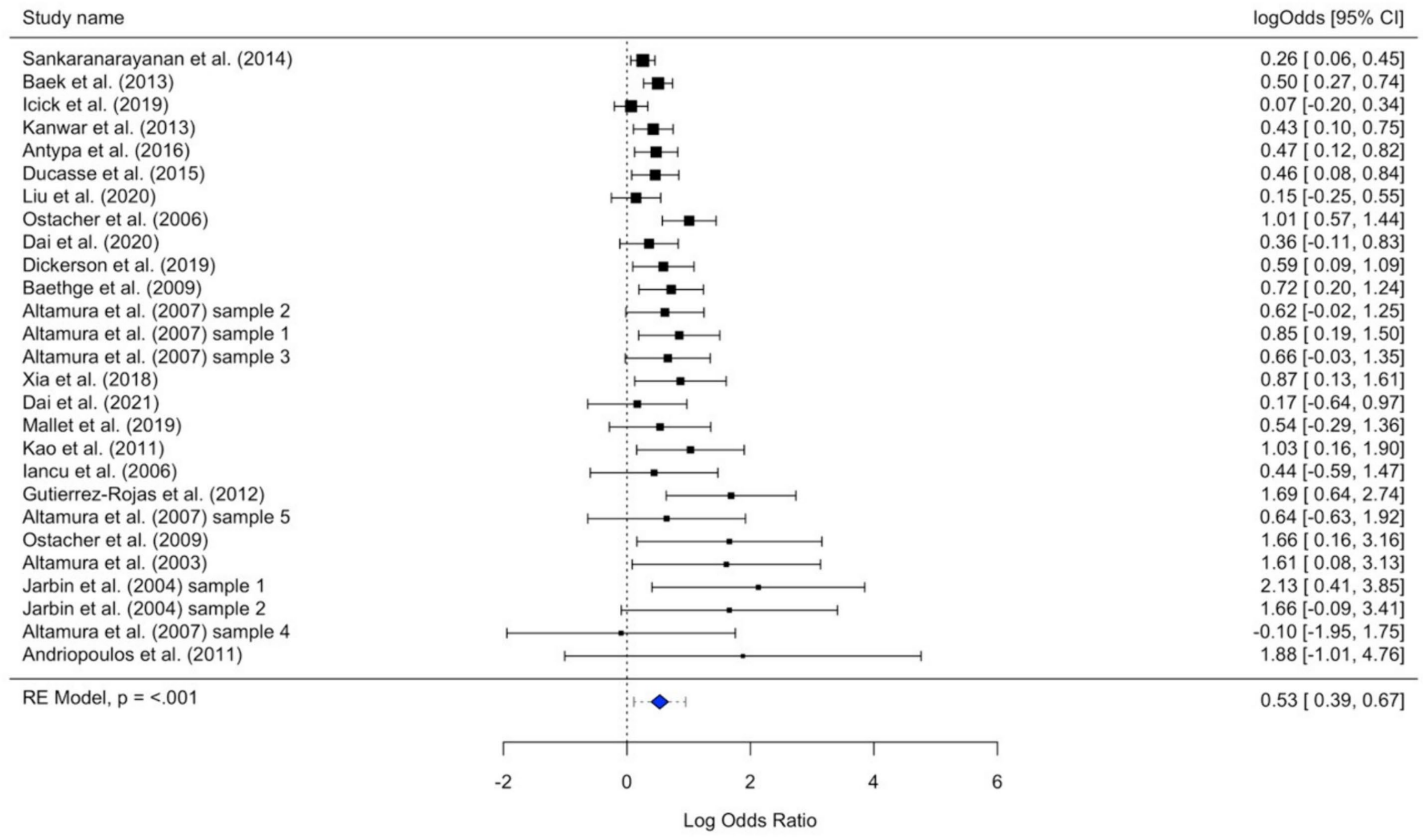
Forest plot of all included studies according to study precision. Effect sizes are provided in log odds ratios with 95% confidence intervals (CI). Symbol size varies according to study precision, with larger squares and shorter whiskers indicating higher study precision.

We observed a small association between smoking and lifetime suicide attempts, indicating larger odds of smokers than non-smokers of lifetime suicide attempts (*OR* = 1.70, 95% *CI* [1.48; 1.95], *p* < 0.001; Figure 2). There was some evidence for non-trivial, albeit small-to-moderate, between-studies heterogeneity (*I*^2^ = 40.28), which indicates effects of moderating variables as a source of the observed differences.

Therefore, we repeated our analyses while excluding two studies that had categorized smokers vs. non-smokers in a different manner than the remaining studies (i.e., most studies categorized past smokers into the non-smoker group while (Ostacher et al., 2006; Icick et al., 2019) categorized them as smokers). However, results were virtually identical, indicating no substantial effect of differing group assignments within primary studies on summary effect estimation (*OR* = 1.65, 95% *CI* [1.46; 1.85], *p* < 0.001). We, therefore, report all subsequent findings based on all *k* = 27 available independent effect sizes (numerical outcomes of these analyses for the subset of *k* = 25 effect sizes were virtually identical).

### 3.1 Subgroup analyses

Effect sizes were differentiated according to study type (*Q* = 12.141, *p* < 0.001), with cohort studies yielding a moderate effect (*OR* = 2.76, *p* < 0.001) that was significantly larger than effects from cross-sectional (*OR* = 1.58, *p* < 0.001) and case–control studies (*OR* = 1.28, *p* = 0.106).

Groups with different diagnoses showed predominantly small non-trivial effects that did not significantly differ between groups (*Q* = 0.864, *p* = 0.353). However, patients with bipolar disorder showed the numerically largest and non-trivial effect (*OR* = 2.02, *p* < 0.001), followed by patients with psychosis (*OR* = 1.64, *p* < 0.001), and the mixed group (*OR* = 1.44, *p* < 0.001).

Similarly, no significant differences between sample types were observed (*Q* = 1.614, *p* = 0.204), yielding mostly non-trivial but small effects. The outpatient group showed numerically somewhat larger effects (*OR* = 1.92, *p* < 0.001) compared to the inpatient (*OR* = 1.67, *p* = 0.008) and the mixed groups (*OR* = 1.42, *p* = 0.002). Numerical results of subgroup summary effects are detailed in Table 2.

**Table 2:** Overall and subgroup-specific summary effects.

	OR	*LBCI*	*UBCI*	*p*	*I* ^**2**^
Overall sample					
All (*k* = 27)	1.70	1.48	1.95	<0.001	40.28%
Diagnosis type					
SCZ (*k* = 16)	1.64	1.39	1.94	<0.001	0.83%
BP (*k* = 8)	2.02	1.42	2.88	<0.001	76.05%
Mixed (*k* = 3)	1.44	1.18	1.75	<0.001	21.11%
Sample type					
Inpatients (*k* = 5)	1.67	1.15	2.42	0.008	58.98%
Outpatients (*k* = 17)	1.92	1.56	2.34	<0.001	36.79%
Mixed (*k* = 5)	1.42	1.13	1.78	0.002	38.57%
Study type					
Cohort (*k* = 6)	2.76	2.03	3.76	<0.001	<0.01%
Cross-sectional (*k* = 16)	1.58	1.41	1.78	<0.001	8.69%
Case–control (*k* = 5)	1.28	0.95	1.71	0.106	26.55%

LBCI, Lower bound of 95% confidence interval; UBCI, Upper bound of 95% confidence interval; SCZ, patients with schizophrenia; BP, patients with bipolar disorder; *I*^2^, true heterogeneity/total observed variation.

### 3.2 Meta-regressions

**Figure 3:**
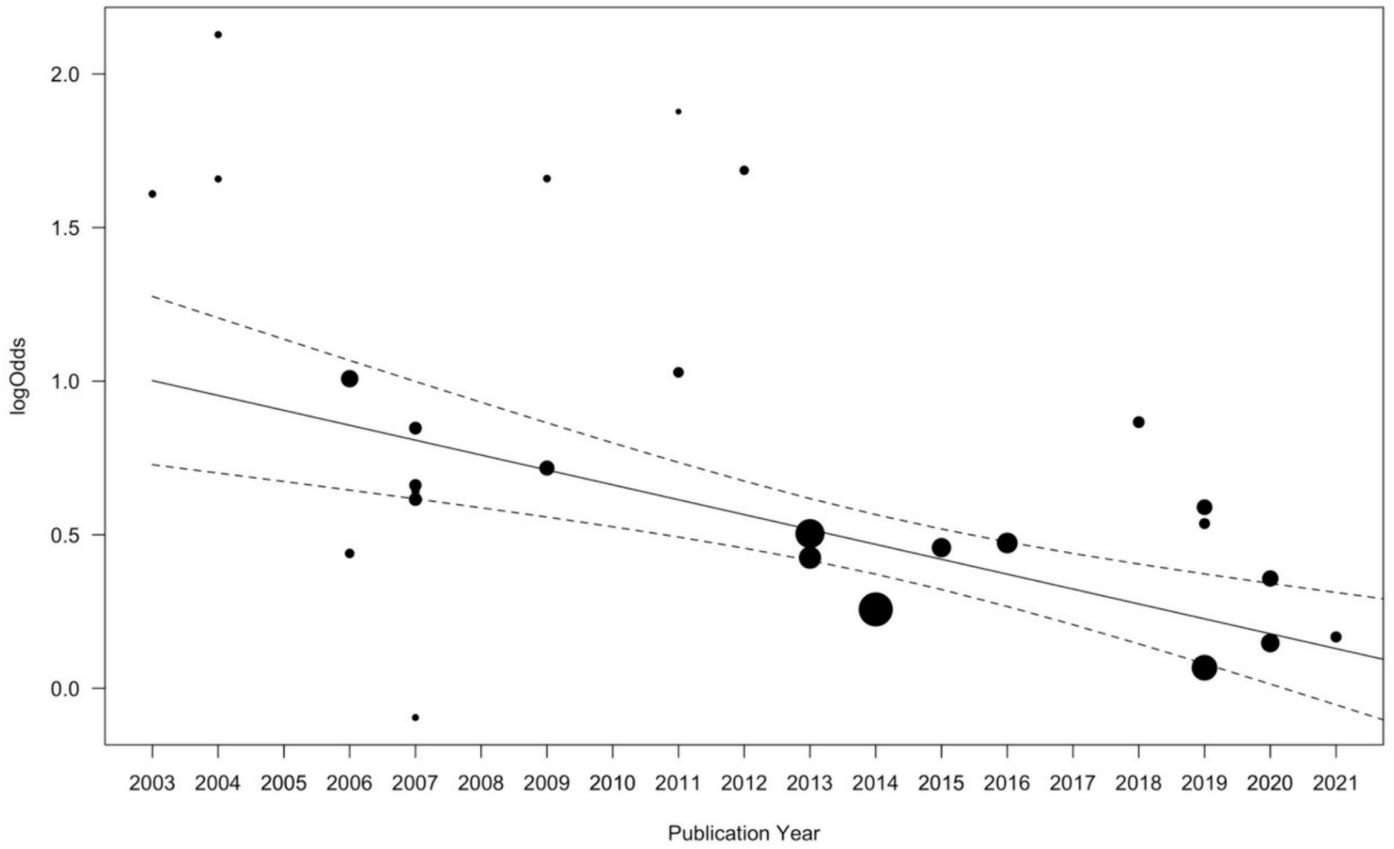
Effects of study publication year on log odds ratios. Symbol size varies according to study precision, with larger bubbles indicating higher study precision. Solid line represents the linear regression; dashed lines represent 95% confidence bands.

**Figure 4:**
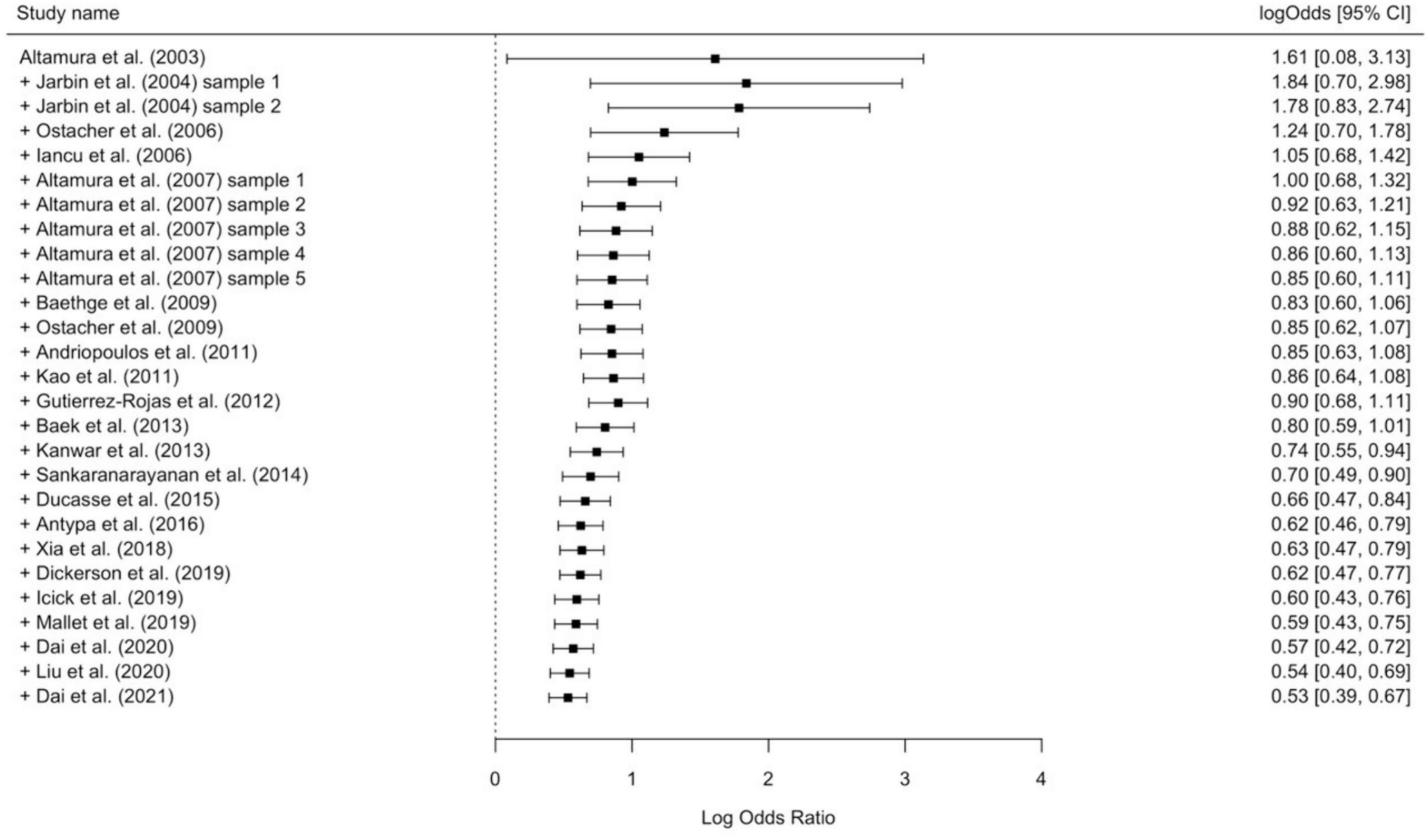
Cumulative forest plot according to study publication year. Effect sizes are provided in log odds ratios with 95% confidence intervals (CI).

A precision-weighted meta-regression of publication year on effect sizes indicated significant decreases in effect strength over time (*b* = −0.097, *Q* = 17.61, *p* < 0.001; Figure 3). Cumulative meta-analyses supported this finding, indicating almost exclusively continuously decreasing summary effect size estimates when individual study effects were added in turn according to study publication years (Figure 4).

**Figure 5:**
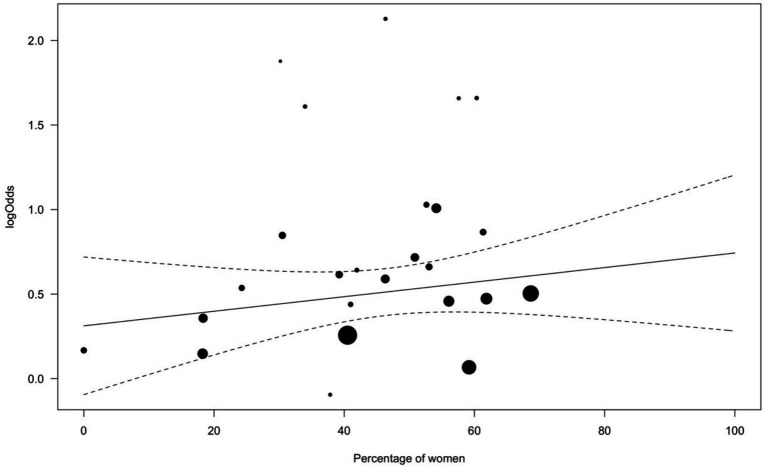
Bubble plot for effects of women percentage within samples on log odds ratios. Symbol size varies according to study precision, with larger bubbles indicating higher study precision. Solid line represents the linear regression; dashed lines represent 95% confidence bands.

In another meta-regression, the percentage of women within samples showed no significant influences on effect sizes (*b* = 0.004, *Q* = 1.05, *p* = 0.305; see Figure 5).

### 3.3 Publication bias

**Figure 6:**
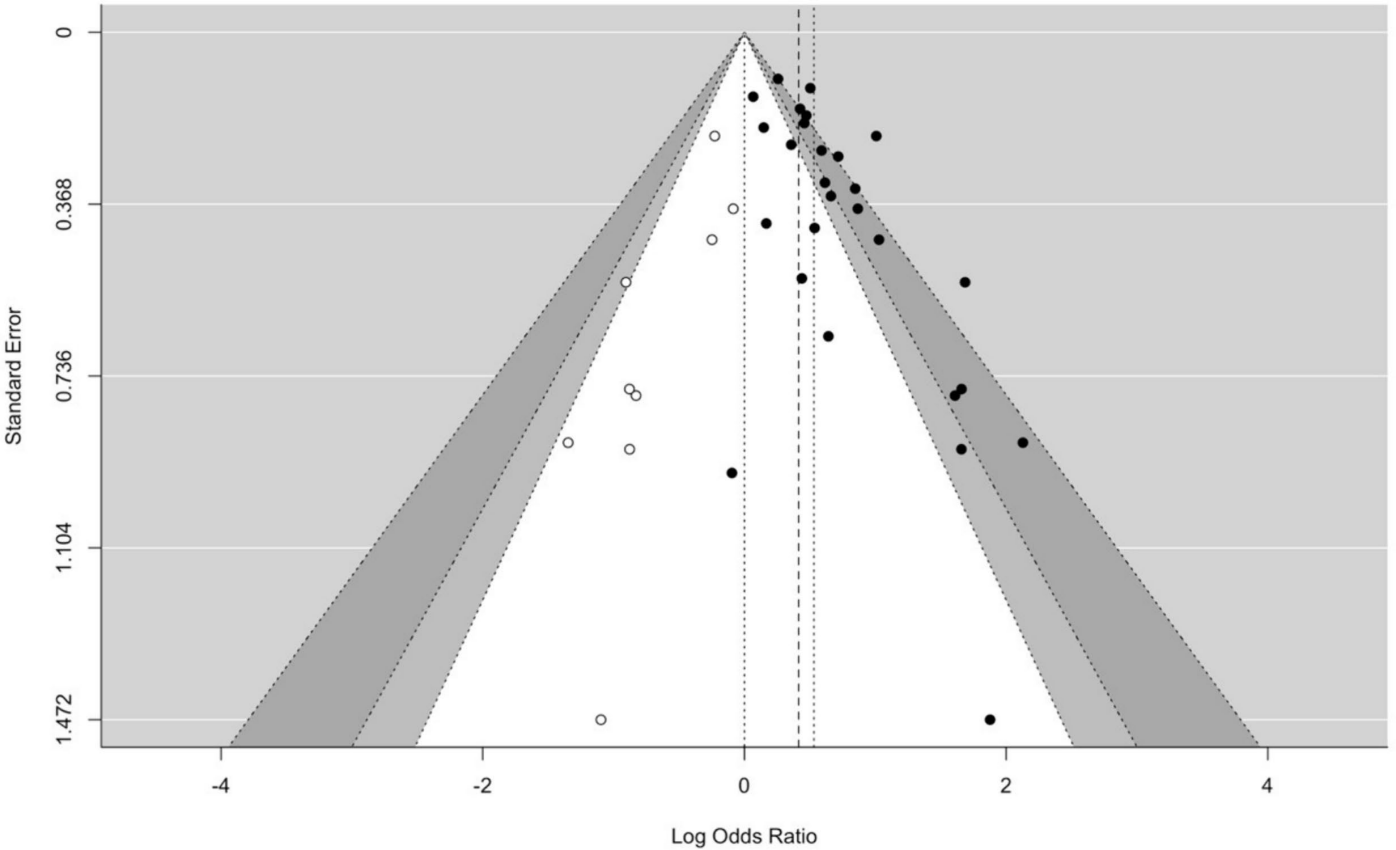
Contour-enhanced funnel plot. Solid dots indicate observed effect sizes; circles indicate effect sizes that were imputed symmetrically to the adjusted effect according to the Trim-and-Fill method. The non-0 vertical lines indicate the observed (dotted) and Trim-and-Fill-based adjusted (dashed) effect.

Visual inspection of the funnel plot showed substantial asymmetry, suggesting inflation of the observed summary effect (Figure 6). Formal tests in terms of Sterne and Egger’s regression method yielded significant results, thus supporting this interpretation (*Z* = 3.81, *p* < 0.001). Results from Trim-and-Fill were consistent with findings from Sterne and Egger’s regression, indicating nine missing effect sizes on the left side of the funnel plot and yielding an adjusted effect of *OR* = 1.51 (Log*OR* = 0.42).

In contrast, the selection model approach did not show evidence for publication bias, indicating an adjusted summary effect that remained within 20% of the value of the observed effect (i.e., *OR* = 1.69 vs. 1.70, respectively).

**Figure 7:**
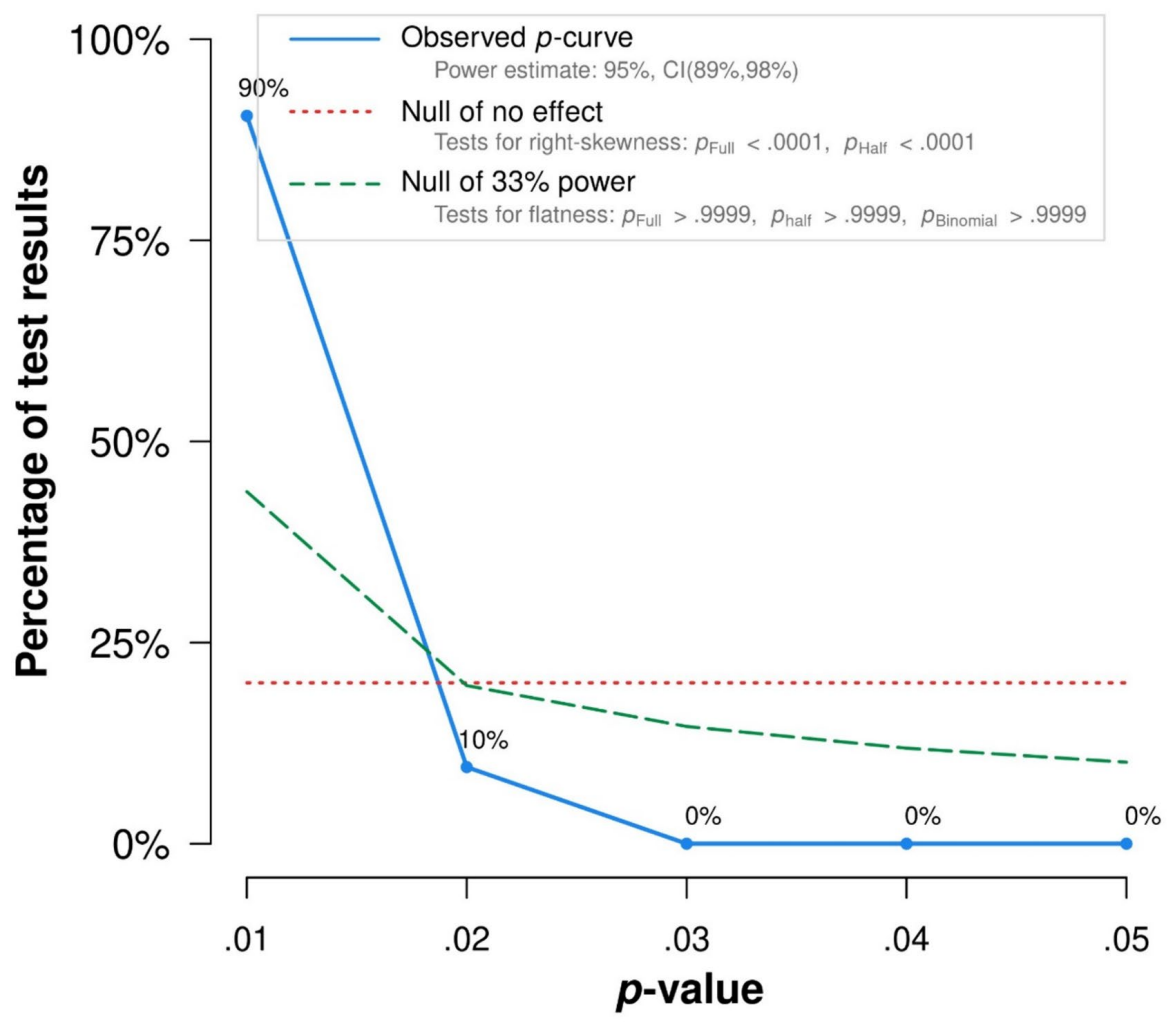
*p*-value distributions of significant values according to *p*-curve. The observed *p*-curve includes 21 statistically significant (*p* < 0.05) results, of which 21 are *p* < 0.025. There were 6 additional results entered but excluded from *p*-curve because they were *p* > 0.05.

There was no significant evidence for *p*-hacking according to *p*-curve analysis in binomial and continuous tests. Moreover, both binomial and continuous tests indicated evidence for an appropriate evidential value of our summary effect calculations (see Figure 7). Similarly, *p*-uniform did not yield evidence for *p*-hacking either (*p* = 0.989). *p*-uniform-based summary effect estimations showed a non-trivial effect, yielding an *OR* = 1.63 (95% *CI* [1.52; 1.75], *p* < 0.001).

## 4 Discussion

In the present meta-analysis, we show a small but meaningful association between smoking and suicide attempts in patients with psychosis and bipolar disorder, indicating larger numbers of lifetime suicide attempts of smoking compared to non-smoking patients. This is important because these findings may contribute to identifying at-risk groups in terms of suicide in mentally ill individuals.

The observed smoking and suicide attempt link generalized over different types of diagnoses (psychosis and bipolar disorder), sample types (in-and outpatients), and participant sex but was differentiated according to study type (i.e., cohort vs. cross-sectional and case–control studies). However, the observed association appears confounded by dissemination bias and shows cross-temporal effect declines, thus indicating that our observed summary effect may be somewhat inflated. Our findings present several points of interest, as we discuss below.

First, in line with a previous meta-analysis (Sankaranarayanan et al., 2015), a patient’s diagnostic group (psychosis vs. bipolar spectrum) did not seem to influence the association between smoking and lifetime suicide attempts. This observed generalizing effect seems plausible because psychosis and bipolar disorder patient groups share comparable (elevated) smoking rates (Dickerson et al., 2013), neurological pathway processing (Kraguljac et al., 2012), and lifetime suicide prevalence (Gonda et al., 2012; Siris, 2001) that differ significantly from general population observations.

However, in contrast to previous findings (Sankaranarayanan et al., 2015), effects appeared to be differentiated according to study type, yielding stronger effects for cohort compared to cross-sectional or case–control studies. Because prospective cohort studies epistemologically offer the most rigorous approach in observational studies (Mann, 2003), this may be interpreted as tentative evidence for effect underestimations in cross-sectional and case–control studies. However, in light of the comparatively small number of included studies, potential alternative reasons for effect differentiation cannot be entirely ruled out.

Second, sample type (in-vs. outpatients) did not significantly influence the observed summary effect. This is somewhat unexpected because being less monitored by potentially aiding and intervening health professionals, especially in the first year of discharge, could arguably be expected to increased suicide attempt prevalence (Chung et al., 2017; Olfson et al., 2016). The more substantial relationship between healthcare satisfaction and subjective quality of life compared to inpatients (Petkari and Pietschnig, 2015) has led researchers to argue that outpatients may be at higher risk of attempting suicide than inpatients. In contrast, other researchers have argued that inpatients might be more prone to attempting suicide due to more severe symptoms, which are the cause for their hospitalization (Bostwick and Pankratz, 2000; Zaheer et al., 2018), as opposed to the protective effects of living independently (Cassidy et al., 2018). In any case, our empirical evidence shows that smoking status and suicide attempt correlations do not appear to be differentiated according to being taken care of in inpatient compared to outpatient settings.

Third, effects generalized across sex, indicating no meaningful influences of sex-specific suicide attempt prevalence with respect to smoking status. This is interesting because women typically show higher depression and suicide attempt prevalence than men, although men typically show a higher prevalence of completed suicides in the general population (Tsirigotis et al., 2011; Värnik, 2012). A similar pattern has been observed in psychotic patients, with women showing larger prevalences of depression and suicide attempts than men (Värnik, 2012; Austad et al., 2015) and men showing a higher prevalence in terms of completed suicides than women (Bertelsen et al., 2007; Healy et al., 2012), although not all patient-based studies were consistent with these results (Lester, 2006; Carlborg et al., 2008). Our results contrast prior findings of differentiated smoking effects on suicide attempts between men and women in young adults of the US general population that indicated positive associations in men, but no effects in women (Zhang et al., 2005). This indicates that smoking status may be useful to identify at-risk groups regardless of patient sex.

Finally, we observed a cross-temporal decline in terms of effect sizes, indicating stronger effects of studies that had been published in earlier years. On the one hand, it is possible that smoking status and suicide attempt associations have genuinely changed in the past decades, conceivably owing to decreasing global smoking [e.g., decreasing sales of cigarettes per adult per day (Forey et al., 2016)] or suicide attempt prevalence (e.g., Xiao et al., 2021, for US data). On the other hand, this observation can be plausibly attributed to the so-called decline effect [i.e., inflated effect sizes having a larger probability of being published earlier which leads to an inflated perception of the true effect in the published scientific literature; (Pietschnig et al., 2019)], thus representing a publication mechanism-related phenomenon that leads to (non-genuine) inflated effects in the published literature. This latter interpretation is supported by evidence for publication bias in funnel-plot asymmetry-based detection methods, which was observed in our present analyses. The presently observed small non-trivial summary effect may thus be considered to represent an upper threshold of the true effect of smoking status and suicide attempt associations in individuals with psychosis and bipolar disorder.

### 4.1 Limitations

Some limitations of this meta-analysis need to be acknowledged. First, due to the correlational design of the present study, it was not possible to empirically establish a causal mechanism in regard to the smoking status and suicide attempt link of individuals with psychosis and bipolar disorder. However, the observed non-trivial link provides a useful means for practical care recommendations. Second, some statistical noise may have been introduced by different design characteristics between primary studies that could not be entirely accounted for in our present meta-analysis (e.g., in terms of different smoking operationalizations or psychosis and bipolar disorder assessments). However, this is common in meta-analytical investigations and was accounted for by modeling between-studies heterogeneity in terms of random-effects calculations. Finally, self-reports of lifetime suicide attempts are susceptible to misclassification (Castelein et al., 2015) and prone to survival bias (only reports by survivors). Nonetheless, suicide attempts have been frequently been observed to predict suicide better than suicide ideation (Millner et al., 2015; May and Klonsky, 2016; Ribeiro et al., 2016). There is no reason to suspect that any of these points may have introduced any systematic influences on summary effect estimations, thus providing evidence for the salience of our observed summary effect.

## 5 Conclusion

Here, we show evidence for a non-trivial association between smoking status and lifetime suicide attempts in individuals with psychosis and bipolar disorder. Smoking patients self-reported significantly larger numbers of lifetime suicide attempts than non-smoking patients, regardless of diagnosis type (i.e., psychosis vs. bipolar spectrum), sample type (i.e., in-vs. outpatients), or sex. This link appears to be small but meaningful, thus suggesting that smoking status represents a useful variable for the identification of at-risk populations for suicide attempts in psychotic patients.

## Data Availability

The original contributions presented in the study are included in the article/supplementary material, further inquiries can be directed to the corresponding author.

## Footnotes

^1^ https://osf.io/rsxhp

## References

[ref9001] Altamura A. C. Bassetti R. Bignotti S. Pioli R. Mundo E. (2003). Clinical variables related to suicide attempts in schizophrenic patients: A retrospective study. Schizophrenia Res. 60, 47–55. doi: 10.1016/S0920-9964(02)00164-0 12505137

[ref9002] Altamura A. C. Mundo E. Bassetti R. Green A. Lindenmayer J. P. Alphs L. . (2007). Transcultural differences in suicide attempters: Analysis on a highrisk population of patients with schizophrenia or schizoaffective disorder. Schizophrenia Res. 89, 140–146. doi: 10.1016/j.schres.2006.08.023 17097854

[ref9003] Andriopoulos I. Ellul J. Skokou M. Beratis S. (2011). Suicidality in the “prodromal”phase of schizophrenia. Compr. Psychiatry 52, 479–485. doi: 10.1016/j.comppsych.2010.10.011 21185016

[ref9004] Antypa N. Souery D. Tomasini M. Albani D. Fusco F. Mendlewicz J. (2016). Clinical and genetic factors associated with suicide in mood disorder patients. Eur Arch Psychiatry Clin Neurosci. 266, 181–193. doi: 10.1007/s00406-015-0658-1 26626456

[ref1] Austad G. Joa I. Johannessen J. O. Larsen T. K. (2015). Gender differences in suicidal behaviour in patients with first-episode psychosis. Early Interv. Psychiatry 9, 300–307. doi: 10.1111/eip.12113, PMID: 24304682

[ref9005] Baek J. H. Eisner L. R. Nierenberg A. A. (2013). Smoking and suicidality in subjects with bipolar disorder: Results from the National Epidemiologic Survey on Alcohol and Related Conditions (NESARC). Depression and Anxiety 30, 982–990. doi: 10.1002/da.22107 23658140

[ref9006] Baethge C. Tondo L. Lepri B. Baldessarini R. J. (2009). Coffee and cigarette use: Association with suicidal acts in 352 Sardinian bipolar disorder patients. Bipolar Disord. 11, 494–503. doi: 10.1111/j.1399-5618.2009.00727.x 19624388

[ref2] Bertelsen M. Jeppesen P. I. Petersen L. Thorup A. Le Quach P. Christensen T. Ø. . (2007). Suicidal behaviour and mortality in first-episode psychosis: the OPUS trial. Br. J. Psychiatry 191, s140–s146. doi: 10.1192/bjp.191.51.s140, PMID: 18055932

[ref3] Bora E. Yucel M. Pantelis C. (2009). Cognitive functioning in schizophrenia, schizoaffective disorder and affective psychoses: Meta-analytic study. Br. J. Psychiatry 195, 475–482. doi: 10.1192/bjp.bp.108.055731, PMID: 19949193

[ref4] Bostwick J. M. Pankratz V. S. (2000). Affective disorders and suicide risk: a reexamination. Am. J. Psychiatry 157, 1925–1932. doi: 10.1176/appi.ajp.157.12.1925 11097952

[ref5] Carlborg A. Jokinen J. Jönsson E. G. Nordström A. L. Nordström P. (2008). Long-term suicide risk in schizophrenia spectrum psychoses: survival analysis by gender. Arch. Suicide Res. 12, 347–351. doi: 10.1080/13811110802325133, PMID: 18828038

[ref6] Cassidy R. M. Yang F. Kapczinski F. Passos I. C. (2018). Risk factors for suicidality in patients with schizophrenia: a systematic review, meta-analysis, and meta-regression of 96 studies. Schizophr. Bull. 44, 787–797. doi: 10.1093/schbul/sbx131, PMID: 29036388 PMC6007264

[ref7] Castelein S. Liemburg E. J. de Lange J. S. van Es F. D. Visser E. Aleman A. . (2015). Suicide in recent onset psychosis revisited: significant reduction of suicide rate over the last two decades—a replication study of a Dutch incidence cohort. PLoS One 10:e0129263. doi: 10.1371/journal.pone.0129263, PMID: 26068417 PMC4466318

[ref8] Chung D. T. Ryan C. J. Hadzi-Pavlovic D. Singh S. P. Stanton C. Large M. M. (2017). Suicide rates after discharge from psychiatric facilities: a systematic review and meta-analysis. JAMA Psychiatry 74, 694–702. doi: 10.1001/jamapsychiatry.2017.1044, PMID: 28564699 PMC5710249

[ref9] Coburn K. M. Vevea J. L. Coburn M. K. (2019). Package ‘weightr’. Estimating weight-function models for publication bias.

[ref10] Cohen J. (1988). Statistical power analysis for the behavioral sciences. 2nd Edn. Mahwah, NJ: L. Erlbaum Associates.

[ref9007] Dai Q. Wang D. Wang J. Xu H. Andriescue E. C. Wu H. E. (2020). Suicide attempts in Chinese Han patients with schizophrenia: Cognitive, demographic, and clinical variables. Braz. J. Psychiatry. 43, 29–34. doi: 10.1590/1516-4446-2020-0900 32401875 PMC7861187

[ref9008] Dai Q. Zhou Y. Liu Y. Wei S. Zhou H. Tian Y. . (2021). Alcohol use history increases the likelihood of suicide behavior among male chronic patients with schizophrenia in a Chinese population. Suicide Life Threat Behav. 52, 716–724. doi: 10.1111/sltb.12855 35318712

[ref11] de Beaurepaire R. Rat P. Beauverie P. Houery M. Niel P. Castéra S. . (2012). Is smoking linked to positive symptoms in acutely ill psychiatric patients? Nord. J. Psychiatry 66, 225–231. doi: 10.3109/08039488.2011.610468, PMID: 21905972

[ref12] Dickerson F. Stallings C. R. Origoni A. E. Vaughan C. Khushalani S. Schroeder J. . (2013). Cigarette smoking among persons with schizophrenia or bipolar disorder in routine clinical settings, 1999-2011. Psychiatr. Serv. 64, 44–50. doi: 10.1176/appi.ps.201200143 23280457

[ref9009] Dickerson F. Stallings C. Origoni A. Katsafanas E. Sweeney K. Khushalani S. (2019). Nitrated meat products are associated with suicide behavior in psychiatric patients. Psychiatry Research 275, 283–286. doi: 10.1016/j.psychres.2019.03.047 30952072

[ref13] Dowle M. Srinivasan A. Gorecki J. Chirico M. Stetsenko P. Short T. . (2019). Package ‘data. table’. Extension of ‘data. frames.

[ref9010] Ducasse D. Jaussent I. Guillaume S. Azorin J. M. Bellivier F. Belzeaux R. . (2015). Increased risk of suicide attempt in bipolar patients with severe tobacco dependence. J. Affect. Disord. 183, 113–118. doi: 10.1016/j.jad.2015.04.038 26001671

[ref14] Dürlinger F. Pietschnig J. (2022). Meta-analyzing intelligence and religiosity associations: evidence from the multiverse. PLoS One 17:e0262699. doi: 10.1371/journal.pone.0262699, PMID: 35148316 PMC8836311

[ref15] Duval S. Tweedie R. (2000). A nonparametric “trim and fill” method of accounting for publication bias in meta-analysis. J. Am. Stat. Assoc. 95, 89–98. doi: 10.1080/01621459.2000.10473905

[ref16] Egger M. Smith G. D. Schneider M. Minder C. (1997). Bias in meta-analysis detected by a simple, graphical test. BMJ 315, 629–634. doi: 10.1136/bmj.315.7109.629, PMID: 9310563 PMC2127453

[ref17] Forey B. Hamling J. Lee P. Wald N. (2016). International smoking statistics: WEB edition. Sutton: P. N. Lee Statistics and Computing Ltd.

[ref18] Gil S. M. Metherate R. (2019). Enhanced sensory-cognitive processing by activation of nicotinic acetylcholine receptors. Nicotine Tob. Res. 21, 377–382. doi: 10.1093/ntr/nty134, PMID: 30137439 PMC6379024

[ref19] Gonda X. Pompili M. Serafini G. Montebovi F. Campi S. Dome P. . (2012). Suicidal behavior in bipolar disorder: epidemiology, characteristics and major risk factors. J. Affect. Disord. 143, 16–26. doi: 10.1016/j.jad.2012.04.041 22763038

[ref20] Green M. F. Horan W. P. Lee J. (2015). Social cognition in schizophrenia. Nat. Rev. Neurosci. 16, 620–631. doi: 10.1038/nrn4005 26373471

[ref21] Gurillo P. Jauhar S. Murray R. M. Mac Cabe J. H. (2015). Does tobacco use cause psychosis? Systematic review and meta-analysis. Lancet Psychiatry 2, 718–725. doi: 10.1016/S2215-0366(15)00152-2, PMID: 26249303 PMC4698800

[ref9011] Gutiérrez-Rojas L. Gurpegui M. Soto F. Martínez-Ortega J. Jurado L. (2012). High nicotine dependence is a factor in the risk of suicide in bipolar disorder patients: Int. Clin. Psychopharmacol. 28, e18–e19. doi: 10.1097/01.yic.0000423262.48950.39

[ref22] Hartz S. M. Pato C. N. Medeiros H. Cavazos-Rehg P. Sobell J. L. Knowles J. A. . (2014). Comorbidity of severe psychotic disorders with measures of substance use. JAMA. Psychiatry 71, 248–254. doi: 10.1001/jamapsychiatry.2013.3726 PMC4060740 24382686

[ref23] Haustein K. O. Haffner S. Woodcock B. G. (2002). A review of the pharmacological and psychopharmacological aspects of smoking and smoking cessation in psychiatric patients. Int. J. Clin. Pharmacol. Ther. 40, 404–418. doi: 10.5414/CPP40404 12358157

[ref24] Healy D. Le Noury J. Harris M. Butt M. Linden S. Whitaker C. . (2012). Mortality in schizophrenia and related psychoses: data from two cohorts, 1875-1924 and 1994-2010. BMJ Open 2:e001810. doi: 10.1136/bmjopen-2012-001810 PMC3488735 23048063

[ref25] Higgins J. P. Thompson S. G. Deeks J. J. Altman D. G. (2003). Measuring inconsistency in meta-analyses. BMJ 327, 557–560. doi: 10.1136/bmj.327.7414.557, PMID: 12958120 PMC192859

[ref9012] Iancu I. Sapir A. P. Shaked G. Poreh A. Dannon P. N. Chelben J. . (2006). Increased Suicidal Risk Among Smoking Schizophrenia Patients. Clin. Neuropharmacol. 29, 230–237. doi: 10.1097/01.WNF.0000228178.75711.EB 16855425

[ref26] Icick R. Melle I. Etain B. Ringen P. A. Aminoff S. R. Leboyer M. . (2019). Tobacco smoking and other substance use disorders associated with recurrent suicide attempts in bipolar disorder. J. Affect. Disord. 256, 348–357. doi: 10.1016/j.jad.2019.05.075, PMID: 31202989

[ref27] Isuru A. Rajasuriya M. (2019). Tobacco smoking and schizophrenia: re-examining the evidence. BJPsych. Adv. 25, 363–372. doi: 10.1192/bja.2019.33

[ref9013] Jarbin H. von Knorring A.-L. (2004). Suicide and suicide attempts in adolescent-onset psychotic disorders. Nord. J. Psychiatry 58, 115–123. doi: 10.1080/08039480410005611 15204217

[ref9014] Kanwar J. Okusaga O. Giegling I. Konte B. Vaswani D. Sleemi A. . (2013). In patients with schizophrenia, non-fatal suicidal selfdirected violence is positively associated with present but not past smoking. Schizophr. Res. 149, 194–195. doi: 10.1016/j.schres.2013.06.010 23810528

[ref9015] Kao Y.-C. Liu Y.-P. Cheng T.-H. Chou M.-K. (2011). Cigarette smoking in outpatients with chronic schizophrenia in Taiwan: Relationships to socio-demographic and clinical characteristics. Psychiatry Res. 190, 193–199. doi: 10.1016/j.psychres.2011.05.016 21621853

[ref28] Khokhar J. Y. Dwiel L. L. Henricks A. M. Doucette W. T. Green A. I. (2018). The link between schizophrenia and substance use disorder: a unifying hypothesis. Schizophr. Res. 194, 78–85. doi: 10.1016/j.schres.2017.04.016, PMID: 28416205 PMC6094954

[ref29] Knapp F. Viechtbauer W. Leonhart R. Nitschke K. Kaller C. P. (2017). Planning performance in schizophrenia patients: a meta-analysis of the influence of task difficulty and clinical and sociodemographic variables. Psychol. Med. 47, 2002–2016. doi: 10.1017/S0033291717000459, PMID: 28385166

[ref30] Kraguljac N. V. Reid M. White D. Jones R. den Hollander J. Lowman D. . (2012). Neurometabolites in schizophrenia and bipolar disorder—a systematic review and meta-analysis. Psychiatry Res. Neuroimaging 203, 111–125. doi: 10.1016/j.pscychresns.2012.02.003, PMID: 22981426 PMC3466386

[ref31] Kumari V. Postma P. (2005). Nicotine use in schizophrenia: the self-medication hypotheses. Neurosci. Biobehav. Rev. 29, 1021–1034. doi: 10.1016/j.neubiorev.2005.02.006, PMID: 15964073

[ref32] Lee P. Van Meter A. (2020). Emotional body language: social cognition deficits in bipolar disorder. J. Affect. Disord. 272, 231–238. doi: 10.1016/j.jad.2020.03.114, PMID: 32553363

[ref33] Lester D. (2006). Sex differences in completed suicide by schizophrenic patients: a meta-analysis. Suicide Life Threat. Behav. 36, 50–56. doi: 10.1521/suli.2006.36.1.50, PMID: 16676625

[ref9016] Liu J. H. Zhu C. Zheng K. Tang W. Gao L. L. Trihn T. H. . (2020). MTHFR Ala222Val polymorphism and clinical characteristics confer susceptibility to suicide attempt in chronic patients with schizophrenia. Scientific Reports 10:5008. doi: 10.1038/s41598-020-57411-1 32193498 PMC7081211

[ref34] Lüdecke D. Lüdecke M. D. David B. W. (2019). Package ‘esc’. R Package Version 0.5.2019.

[ref9017] Mallet J. Strat J. L. Schürhoff F. Mazer N. Portalier C. Andrianarisoa M. . (2019). Tobacco smoking is associated with antipsychotic medication, physical aggressiveness, and alcohol use disorder in schizophrenia: results from the FACE-SZ national cohort. Eur Arch Psychiatry Clin Neurosci. 269, 449–457. doi: 10.1007/s00406-018-0873-7 29396753

[ref35] Mann C. J. (2003). Observational research methods. Research design II: cohort, cross sectional, and case-control studies. Emerg. Med. J. 20, 54–60. doi: 10.1136/emj.20.1.54, PMID: 12533370 PMC1726024

[ref36] Martin L. M. Sayette M. A. (2018). A review of the effects of nicotine on social functioning. Exp. Clin. Psychopharmacol. 26, 425–439. doi: 10.1037/pha0000208, PMID: 29952615 PMC6162172

[ref37] May A. M. Klonsky E. D. (2016). What distinguishes suicide attempters from suicide ideators? A meta-analysis of potential factors. Clin. Psychol. Sci. Pract. 23, 5–20. doi: 10.1111/cpsp.12136

[ref38] Millner A. J. Lee M. D. Nock M. K. (2015). Single-item measurement of suicidal behaviors: validity and consequences of misclassification. PLoS One 10:e0141606. doi: 10.1371/journal.pone.0141606, PMID: 26496707 PMC4619664

[ref39] Miskowiak K. W. Seeberg I. Kjaerstad H. L. Burdick K. E. Martinez-Aran A. del Mar B. C. . (2019). Affective cognition in bipolar disorder: a systematic review by the ISBD targeting cognition task force. Bipolar Disord. 21, 686–719. doi: 10.1111/bdi.12834, PMID: 31491048

[ref40] Olfson M. Wall M. Wang S. Crystal S. Liu S. M. Gerhard T. . (2016). Short-term suicide risk after psychiatric hospital discharge. JAMA Psychiatry 73, 1119–1126. doi: 10.1001/jamapsychiatry.2016.2035, PMID: 27654151 PMC8259698

[ref41] Ostacher M. J. Nierenberg A. A. Perlis R. H. Eidelman P. Borrelli D. J. Tran T. B. . (2006). The relationship between smoking and suicidal behavior, comorbidity, and course of illness in bipolar disorder. J. Clin. Psychiatry 67, 1907–1911. doi: 10.4088/JCP.v67n1210, PMID: 17194268

[ref9018] Ostacher M. J. LeBeau R. T. Perlis R. H. Nierenberg A. A. Lund H. G. Moshier S. J. . (2009). Cigarette smoking is associated with suicidality in bipolar disorder. Bipolar Disord. 11, 766–771. doi: 10.1111/j.1399-5618.2009.00744.x 19840000 PMC2918237

[ref42] Petkari E. Pietschnig J. (2015). Associations of quality of life with service satisfaction in psychotic patients: a meta-analysis. PLoS One 10:e0135267. doi: 10.1371/journal.pone.0135267, PMID: 26275139 PMC4537198

[ref43] Pietschnig J. Siegel M. Eder J. S. Gittler G. (2019). Effect declines are systematic, strong, and ubiquitous: a meta-meta-analysis of the decline effect in intelligence research. Front. Psychol. 10:2874. doi: 10.3389/fpsyg.2019.02874, PMID: 31920891 PMC6930891

[ref44] Pompili M. Gonda X. Serafini G. Innamorati M. Sher L. Amore M. . (2013). Epidemiology of suicide in bipolar disorders: a systematic review of the literature. Bipolar Disord. 15, 457–490. doi: 10.1111/bdi.12087 23755739

[ref45] R Core Team (2023). R: A language and environment for statistical computing. Vienna: R Foundation for Statistical Computing.

[ref46] Ribeiro J. D. Franklin J. C. Fox K. R. Bentley K. H. Kleiman E. M. Chang B. P. . (2016). Self-injurious thoughts and behaviors as risk factors for future suicide ideation, attempts, and death: a meta-analysis of longitudinal studies. Psychol. Med. 46, 225–236. doi: 10.1017/S0033291715001804, PMID: 26370729 PMC4774896

[ref9019] Sankaranarayanan A. Mancuso S. Castle D. (2014). Smoking and suicidality in patients with a psychotic disorder. Psychiatry Res. 215, 634–640. doi: 10.1016/j.psychres.2013.12.032 24411712

[ref47] Sankaranarayanan A. Mancuso S. Wilding H. Ghuloum S. Castle D. (2015). Smoking, suicidality and psychosis: a systematic meta-analysis. PLoS One 10:e0138147. doi: 10.1371/journal.pone.0138147, PMID: 26372218 PMC4570823

[ref48] Savla G. N. Vella L. Armstrong C. C. Penn D. L. Twamley E. W. (2013). Deficits in domains of social cognition in schizophrenia: a meta-analysis of the empirical evidence. Schizophr. Bull. 39, 979–992. doi: 10.1093/schbul/sbs080, PMID: 22949733 PMC3756768

[ref49] Siegel M. Eder J. S. Wicherts J. M. Pietschnig J. (2022). Times are changing, bias isn’t: a meta-meta-analysis on publication bias detection practices, prevalence rates, and predictors in industrial/organizational psychology. J. Appl. Psychol. 107, 2013–2039. doi: 10.1037/apl0000991, PMID: 34968082

[ref50] Simonsohn U. Nelson L. D. Simmons J. P. (2014). P-curve and effect size: correcting for publication bias using only significant results. Perspect. Psychol. Sci. 9, 666–681. doi: 10.1177/1745691614553988, PMID: 26186117

[ref51] Siris S. G. (2001). Suicide and schizophrenia. J. Psychopharmacol. 15, 127–135. doi: 10.1177/026988110101500209 11448086

[ref52] Tsirigotis K. Gruszczynski W. Tsirigotis M. (2011). Gender differentiation in methods of suicide attempts. Medical science monitor: international medical journal of experimental and clinical. Research 17, PH65–PH70. doi: 10.12659/MSM.881887 PMC3539603 21804473

[ref53] van Aert R. C. van Aert M. R. van Assen V. A. (2020). Package “puniform.”.

[ref54] Van Assen M. A. van Aert R. Wicherts J. M. (2015). Meta-analysis using effect size distributions of only statistically significant studies. Psychol. Methods 20, 293–309. doi: 10.1037/met0000025 25401773

[ref55] Värnik P. (2012). Suicide in the world. Int. J. Environ. Res. Public Health 9, 760–771. doi: 10.3390/ijerph9030760, PMID: 22690161 PMC3367275

[ref56] Vevea J. L. Woods C. M. (2005). Publication bias in research synthesis: sensitivity analysis using a priori weight functions. Psychol. Methods 10, 428–443. doi: 10.1037/1082-989X.10.4.428, PMID: 16392998

[ref57] Viechtbauer W. (2010). Conducting meta-analyses in R with the metafor package. J. Stat. Softw. 36, 1–48. doi: 10.18637/jss.v036.i03

[ref58] Wells G. A. Shea B. O’Connel D. Peterson J. Welch V. Losos M. . (2021). The Newcastle-Ottowa scale (NOS) for assessing the quality of nonrandomized studies in meta-analyses. Ottowa: Ottowa Hospital Research Institute.

[ref59] Wickham H. Bryan J. Kalicinski M. Valery K. Leitienne C. Colbert B. . (2019). Package ‘readxl’. Version, 1.3.

[ref60] Wickham H. Wickham M. H. (2017). Package tidyverse. Easily install and load the ‘Tidyverse.

[ref9020] Xia H. Zhang G. Du X. Zhang Y. Yin G. Dai J. . (2018). Suicide attempt, clinical correlates, and BDNF Val66Met polymorphism in chronic patients with schizophrenia. Neuropsychol. 32, 199–205. doi: 10.1037/neu0000383 28857598

[ref61] Xiao Y. Cerel J. Mann J. J. (2021). Temporal trends in suicidal ideation and attempts among US adolescents by sex and race/ethnicity, 1991-2019. JAMA Netw. Open 4:e2113513. doi: 10.1001/jamanetworkopen.2021.13513, PMID: 34125218 PMC8204211

[ref62] Zaheer J. Jacob B. de Oliveira C. Rudoler D. Juda A. Kurdyak P. (2018). Service utilization and suicide among people with schizophrenia spectrum disorders. Schizophr. Res. 202, 347–353. doi: 10.1016/j.schres.2018.06.025 29935885

[ref63] Zhang J. McKeown R. E. Hussey J. R. Thompson S. J. Woods J. R. (2005). Gender differences in risk factors for attempted suicide among young adults: findings from the third National Health and nutrition examination survey. Ann. Epidemiol. 15, 167–174. doi: 10.1016/j.annepidem.2004.07.095, PMID: 15652723

